# Gut microbiota in gastric cancer: from pathogenesis to precision medicine

**DOI:** 10.3389/fmicb.2025.1606924

**Published:** 2025-07-30

**Authors:** Shuang Huo, Kaiying Lv, Lutuo Han, Yu Zhao, Jiakang Jiang

**Affiliations:** ^1^Graduate School, Heilongjiang University of Chinese Medicine, Harbin, China; ^2^Department of Oncology, First Affiliated Hospital of Heilongjiang University of Chinese Medicine, Harbin, China

**Keywords:** gut microbiota, gastric cancer, dysbiosis, tumor microenvironment, *Helicobacter pylori*, inflammation

## Abstract

Gastric cancer (GC) remains a significant global health burden, driven by a complex interplay of genetic, environmental, and microbial factors. Emerging evidence highlights the critical role of gut microbiota in gastric carcinogenesis, as microbial dysbiosis disrupts gastrointestinal homeostasis, fuels chronic inflammation, and promotes immunomodulation and metabolic reprogramming. *Helicobacter pylori*, a key microbial player, initiates tumorigenic pathways through reactive oxygen species production and the manipulation of dietary and microbial metabolites, leading to epigenetic and genetic alterations. Targeting gut microbiota has emerged as a promising therapeutic strategy, with interventions such as probiotics, prebiotics, dietary modifications, antibiotics, and fecal microbiota transplantation (FMT) showing potential in restoring microbial balance and attenuating tumor progression. Furthermore, advances in microbiota research have identified microbial biomarkers as valuable tools for early diagnosis, prognosis, and personalized treatment of GC. This review evaluates therapeutic strategies for microbiota modulation, assesses its diagnostic and prognostic potential, and highlights current gaps in the field. It also advocates for the integration of microbiota-targeted therapies into clinical practice, emphasizing their transformative potential in the prevention and management of GC. By addressing these aspects, this review aims to provide a comprehensive understanding of the role of gut microbiota in GC and to guide future research and clinical applications.

## 1 Introduction

Gastric cancer (GC) remains a significant public health challenge, ranking as the third most common cancer and the fifth leading cause of cancer-related deaths. Annually, over a million new cases are diagnosed, with more than 700,000 deaths, predominantly in regions such as East Asia, Eastern Europe, and parts of South America (Yang et al., 2020). Despite advancements in diagnostics and treatments, the prognosis for advanced GC remains poor, with a 5-year survival rate of 30%, largely due to late-stage detection and treatment resistance (Sexton et al., 2020). This highlights the urgent need for innovative strategies in prevention, early diagnosis, and effective management of GC.

The human gut microbiota, composed of trillions of microorganisms, plays a pivotal role in maintaining digestive health and overall well-being. Beyond its functions in nutrient digestion, immune regulation, and gut barrier integrity (Takiishi et al., 2017), the imbalance of the gut microbiota, known as dysbiosis, has been increasingly implicated in the development and progression of GC (La Rosa et al., 2020). *Helicobacter pylori* (*H. pylori*), classified as a Group 1 carcinogen by the International Agency for Research on Cancer, is a primary driver of GC through mechanism such as chronic inflammation, genomic instability, and DNA methylation (Yang et al., 2021). Other gut bacteria, such as *Fusobacterium nucleatum* and *pks^+^ Escherichia coli*, along with their byproducts like nitrosamines and reactive oxygen species (ROS), further contribute to a tumor-promoting microenvironment (Liu et al., 2021; Zhang W. et al., 2023; Udayasuryan et al., 2024). The gut microbiota also influences the tumor microenvironment (TME) by modulating immune responses and metabolic pathways, while dysbiosis can impair the efficacy of treatments such as chemotherapy and immune checkpoint inhibitors (Miller and Carson, 2020; Luu et al., 2023).

Emerging research underscores the potential of gut microbiota as a biomarker for early GC detection and risk stratification. Non-invasive methods, including stool and saliva microbiota analysis, have identified specific microbial signatures associated with GC progression, such as elevated *Akkermansia muciniphila* and reduced diversity of beneficial bacteria (Ghaffari et al., 2023). Therapeutic strategies targeting the gut microbiome, such as probiotics, prebiotics, and fecal microbiota transplantation (FMT), have shown promise in restoring microbial balance, reducing inflammation, and inhibiting tumor growth (Keikha and Karbalaei, 2021; Zhao and Jiang, 2021). However, challenges remain, including the variability of individual microbiota compositions influenced by genetics, diet, and environment, as well as ethical and safety concerns related to treatments like FMT. Addressing these issues requires interdisciplinary collaboration to translate findings into effective clinical applications. This review explores the complex relationship between gut microbiota and GC, highlighting its role in carcinogenesis, therapeutic potential, and diagnostic utility, with the aim of informing future strategies for GC prevention and treatment.

## 2 The gut microbiota in health and disease

The gut microbiota is a dynamic and diverse community of trillions of microorganisms, including bacteria, archaea, viruses, fungi, and other microbes, residing in the human digestive tract (Wang et al., 2022). This complex ecosystem is present in all individuals, regardless of health status, though its role in disease states has been the focus of extensive research due to its profound impact on health and pathology. In a healthy state, microbiota plays a critical role in regulating immune responses, metabolism, and protective functions, making it essential for maintaining overall health. *Firmicutes* and *Bacteroidetes* are the two dominant phyla, constituting over 90% of the microbiota, while other significant groups, such as *Actinobacteria*, *Proteobacteria*, and *Verrucomicrobia*, contribute to host physiology and maintain microbial balance (Mestre et al., 2018).

One of the primary functions of gut microbiota is nutrient breakdown. These microorganisms produce enzymes that digest complex carbohydrates, which are otherwise indigestible in the upper gastrointestinal tract. Dietary fibers are fermented into short-chain fatty acids (SCFAs), which serve as an energy source for gut epithelial cells, regulate lipid and glucose metabolism, and exhibit anti-inflammatory properties (Gill et al., 2018; Mann et al., 2024). Additionally, gut microbes synthesize essential vitamins, including vitamin K and B vitamins (B6, B12, and folate), which are crucial for DNA synthesis and cellular metabolism (Uebanso et al., 2020; Pham et al., 2021; Yang et al., 2024).

The gut microbiome also plays a pivotal role in immune regulation. Within the gut-associated lymphoid tissue (GALT), microbial antigens interact with innate immune cells, such as dendritic cells and macrophages, triggering the development of regulatory T cells (Tregs) and the production of secretory immunoglobulin A (IgA) (Pearson et al., 2012; Dadarwal et al., 2017; Bemark et al., 2024). Furthermore, gut bacteria contribute to the integrity of the intestinal barrier by enhancing the production of tight junction proteins, which form a barrier between epithelial cells, preventing the translocation of harmful bacteria and toxins (Ma et al., 2022; Neurath et al., 2025). While the balance of gut bacteria is crucial for health, it can be disrupted by external factors, leading to dysbiosis. This imbalance has significant implications, particularly for gastric health, as explored in the following section.

## 3 Dysbiosis and its impact on gastric health

Dysbiosis refers to the disruption of the gut microbial balance, leading to adverse health effects. Dysbiosis is a critical factor in gastric health, contributing to chronic inflammation, immune dysfunction, and carcinogenesis. Understanding its mechanisms and developing strategies to restore microbial balance could significantly improve the prevention and treatment of GC. Dysbiosis can result from various factors, including diet, antibiotic use, infections, chronic stress, and environmental pollutants (Feng et al., 2020; Alvarez et al., 2021; Mostafavi Abdolmaleky and Zhou, 2024). A Western-style diet, high in fat and sugar but low in fiber, is particularly detrimental. It reduces microbial diversity, promotes the growth of harmful bacteria, and decreases the production of beneficial metabolites such as SCFAs (Malesza et al., 2021).

Chronic atrophic gastritis, a complex syndrome commonly characterized by progressive gastric mucosal atrophy and gland depletion, is a well-studied consequence of dysbiosis and is an important risk factor for GC (Sgambato et al., 2017; Lahner et al., 2020; Conti et al., 2021). Notably, although *H. pylori* is the major driver of gastric inflammation and GC development, other microorganisms such as *Fusobacterium nucleatum* and *Escherichia coli* also play important roles in the formation of inflammatory microenvironment and carcinogenesis through complex interactions that promote the transformation of gastric mucosa from chronic inflammation to malignant (Mima et al., 2017). The production of ROS and other harmful metabolites by these bacteria directly damages gastric DNA, leading to mutations (Shields et al., 2021). Additionally, an overabundance of *Proteobacteria* increases lipopolysaccharides (LPS) production, exacerbating inflammation and impairing immune defenses (Larsen, 2017).

**Figure 1:**
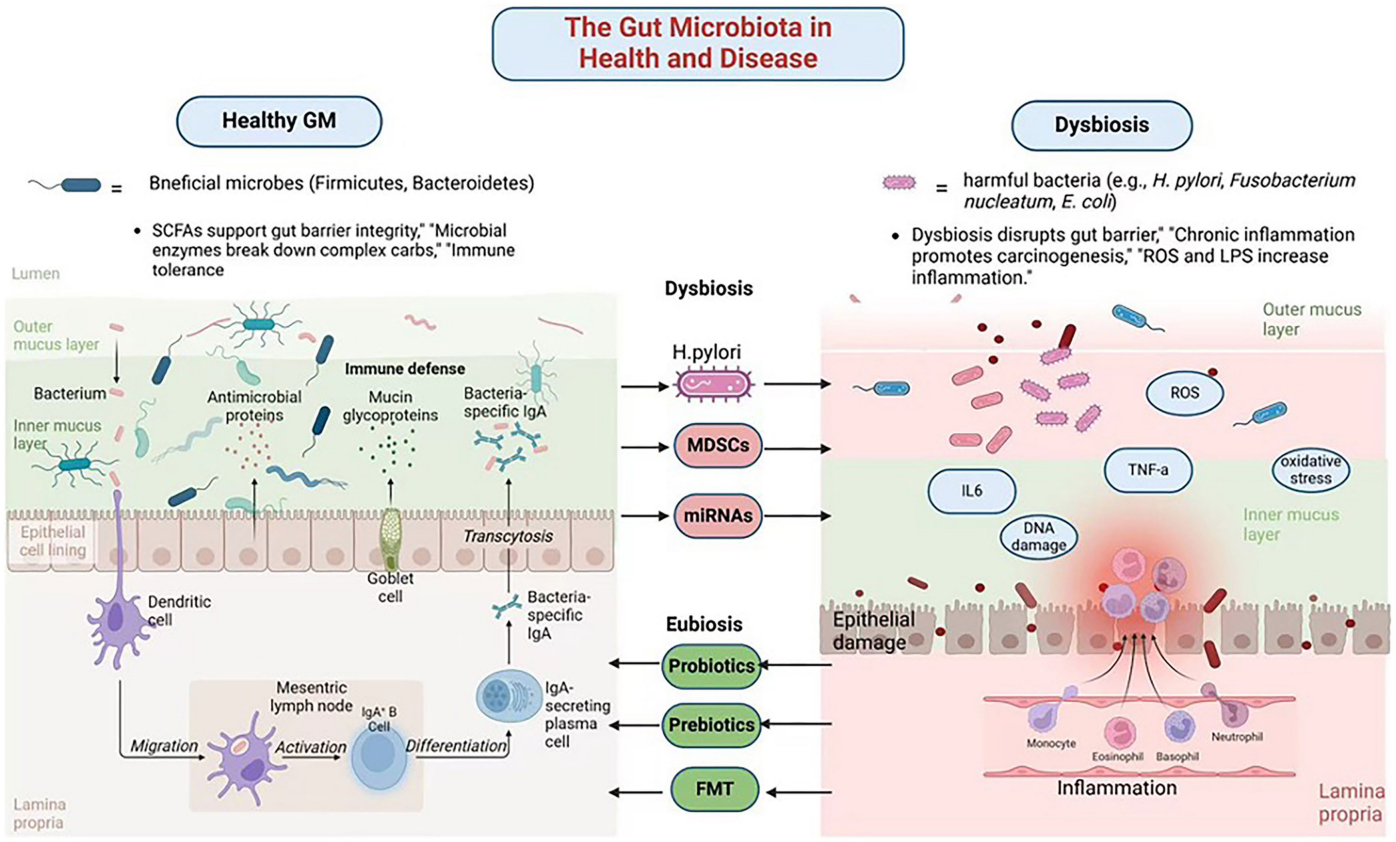
The gut microbiota in health and disease. Created in Biorender.com.

Dysbiosis also contributes to peptic ulcers by reducing the production of butyrate, a key SCFA that maintains the mucus layer protecting gastric epithelial cells (Li et al., 2024). Beyond local gastric effects, dysbiosis alters bile acid metabolism, generating secondary bile acids that further exacerbate gastrointestinal inflammation and carcinogenesis (Jia et al., 2018). Dysbiosis also contributes to immune dysfunction by impairing CD8 + T cell activity and fostering an immunosuppressive TME (Casalegno Garduno and Dabritz, 2021). This is particularly evident in advanced GC cases, where dysbiosis-associated immune alterations typically manifest as elevated levels of immunosuppressive cell populations, including Tregs and myeloid-derived suppressor cells (MDSCs), consequently dampening anti-tumor immune responses (Fan et al., 2020) (Figure 1; Table 1).

**Table 1:** The gut microbiota in health and disease.

Aim of study	Main findings	Conclusions	References
To explore the composition and function of gut microbiota in a healthy individual	*Firmicutes* and *Bacteroidetes* dominate, with *Actinobacteria*, *Proteobacteria*, and *Verrucomicrobia* contributing to metabolism and immune modulation	Balanced microbiota is essential for digestion, immune system regulation, and metabolic homeostasis	Gill et al. (2018)
To understand microbial contributions to vitamin synthesis and nutrient absorption	Gut microbiota produces vitamin K, B6, B12, and folate Supports nutrient absorption and enhances digestive efficiency	Deficiencies in microbiota diversity can lead to vitamin deficiencies and metabolic disorders	Uebanso et al. (2020)
To assess the impact of dysbiosis on gastric health	Dysbiosis leads to chronic inflammation Increased oxidative stress Epithelial barrier disruption, promoting carcinogenesis	Gut microbiota imbalances are strongly linked to GC development and other metabolic diseases	Alvarez et al. (2021)
To evaluate the role of gut microbiota in immune system function	Gut microbiota interacts with GALT Regulates immune responses through microbial metabolites like SCFAs	Microbiota plays a critical role in immune homeostasis, and its imbalance can contribute to inflammatory diseases	Cebra et al. (1998)
To investigate the role of gut microbiota in metabolic regulation	Gut bacteria influence glucose metabolism, lipid breakdown, and amino acid processing SCFAs (butyrate, acetate, propionate) impact energy balance and inflammation	Metabolic dysregulation due to gut microbiota imbalance can contribute to obesity and metabolic disorders	Choi et al. (2021)

## 4 Mechanisms linking gut microbiota to gastric cancer

### 4.1 Chronic inflammation and immune modulation

Chronic inflammation, a hallmark of GC, is significantly influenced by microbial interactions. Virulence proteins such as CagA and VacA from *H. pylori* generate ROS, tumor necrosis factor-alpha (TNF-α), and interleukin-1 beta (IL-1β). These factors collectively induce genomic instability, activate immune cells, and damage epithelial cells, fostering a tumor-promoting environment (Han et al., 2022). However, *H. pylori* is not the sole contributor to GC-related inflammation. Studies reveal that *Fusobacterium nucleatum* activates pathways like NF-κB and STAT3, thereby exacerbating inflammatory responses (Chen et al., 2022). Notably, *Fusobacterium nucleatum* not only drives the recruitment and differentiation of tumor-associated neutrophils (TANs) into pro-tumoral subtypes but also facilitates immune evasion while paradoxically enhancing the efficacy of anti-programmed death-ligand 1 (PD-L1) antibody therapy (Zhang and Pan, 2020). Furthermore, *Fusobacterium nucleatum*-derived extracellular vesicles exacerbate chemoresistance by enhancing oxaliplatin resistance and promoting malignant phenotypes in GC cells (Wei et al., 2022). Similarly, *Escherichia coli* maintains inflammation and promotes epithelial transformation through chronic colonization. It also induces DNA damage and amplifies inflammation cascades, fostering tumorigenesis via genotoxins such as colibactin (Ding et al., 2010; Bossuet-Greif et al., 2018).

Microbial metabolites play a pivotal role in modulating the TEM. SCFAs like butyrate exhibit dual effects depending on the microbial context. Under certain conditions, butyrate reduces autophagic inhibition and cytotoxicity in tumor-associated macrophages (TAMs) by suppressing immunosuppressive molecules such as PD-L1 and IL-10 (Dong et al., 2022). This suppression stimulates cytotoxic T cells, enhancing anti-tumor immune responses. Conversely, metabolites like LPS and ROS activate MDSCs, which suppress anti-tumor immunity and promote an immunosuppressive environment.

The interplay between *H. pylori*, other pathogenic microbes, and immune cell activation underscores the role of chronic inflammation in GC development. Targeting this inflammation through anti-inflammatory probiotics or inhibitors of microbial virulence factors could mitigate cancer-promoting inflammation.

### 4.2 Metabolic reprogramming

The gut microbiota significantly influences metabolic reprogramming, a critical feature of tumor progression in GC. Microbial metabolic activity shapes the TME by altering energy sources and producing metabolites that drive tumor growth and immune evasion. Gut microbes manipulate glucose metabolism, a process central to the Warburg effect observed in many cancers, including GC (Nakagawa et al., 2020). In dysbiosis, tumor cells shift toward aerobic glycolysis, characterized by increased glucose uptake and lactate production. This metabolic shift drives rapid tumor growth and creates an acidic microenvironment that inhibits immune cell activity and facilitates tumor invasion. Microbial metabolites like lactate and succinate contribute to this metabolic reprogramming. Dysbiosis similarly disrupts lipid metabolism, with secondary bile acids from microbial metabolism promoting lipid accumulation in the TME (Sipe et al., 2020). These lipids serve as energy sources for tumor cells and support survival and metastatic pathways. Furthermore, dysbiotic microbial communities impair fatty acid oxidation, disrupting lipid homeostasis and promoting tumorigenesis (Le Noci et al., 2021). For example, arginine depletion, a critical substrate for T cell activation, suppresses immune surveillance and enables tumor cells to evade immune detection (Szefel et al., 2019).

The dynamic interplay between host and microbial metabolic pathways highlights the potential for targeting metabolic reprogramming in GC. Therapeutic strategies aimed at restoring microbial balance or interfering with tumor-promoting metabolites could curb metabolic adaptations that support tumor progression.

### 4.3 Epigenetic and genetic modifications

Emerging research highlights a bidirectional crosstalk between the host and gut microbiota, mediated by the epigenome-microbiome axis, where host epigenotypes dynamically influence gut microbiota composition through transcriptional regulation without altering the genetic code, while microbial metabolites reciprocally drive host epigenetic reprogramming, contributing to gastric carcinogenesis (Pepke et al., 2024a,b). Dysbiotic microbiota induce genotoxic damage through multiple pathways. For instance, pks + *Escherichia coli* synthesizes colibactin, which generates DNA interstrand cross-links and double-strand breaks, activating the ATM/ATR-CHK2 DNA damage response and promoting mutations in TP53 and KRAS (Rahman et al., 2022; Wong and Yu, 2025). Similarly, *H. pylori* generate ROS, causing 8-oxogyanine lesions that drive G to T transversions in CDKN2A/p16 and CDH1 (Hahm et al., 2022; Wu et al., 2023). Additionally, *Fusobacterium nucleatum* exacerbates genomic instability by downregulating mismatch repair proteins MSH2 and MLH1 via ROS, leading to microsatellite instability (Wei et al., 2022; Udayasuryan et al., 2024).

Epigenetic remodeling plays an equally critical role in gastric carcinogenesis, with epigenetic alterations and changes in gene expression serving as key mechanisms (Yang et al., 2023). The *H. pylori* virulence factor CagA hijacks SHP-2 to activate DNMT1 and DNMT3B, inducing hypermethylation of CpG islands in CDKN2A/p16, RPRM, RUNX3, and LOX promoters, thereby establishing a CpG Island Methylator Phenotype (CIMP) in 30–40% of gastric tumors (Kontizas et al., 2020; Bhattacharjee et al., 2024). CagA also recruits p300 to increase H3K27 acetylation at oncogenes such as c-MYC and c-JUN, while VacA promotes repressive H3K9me3 via SUV39H1, silencing tumor suppressors FOXP3 and DUSP5 (Patel et al., 2017). Microbial metabolites exhibit context-dependent effects on the epigenome-microbiome axis: butyrate inhibits histone deacetylases (HDACs I/IIa) to upregulate CDKN1A/p21 and BAX (Cheng et al., 2019; Yao et al., 2024), hydrogen sulfide inhibits HDACs and DNMTs while activating NF-κB through p65 persulfidation (Jones and Neish, 2017), and secondary bile acids (deoxycholate) activate FXR to suppress sFRP1, promoting β-catenin nuclear translocation (Demirkiran et al., 2024). SCFAs, including butyrate, acetate, and propionate derived from microbial polysaccharide fermentation, serve as central regulators of the epigenome-microbiome axis. They inhibit HDACs to prevent chromatin condensation, enhance DNA demethylation by activating ten-eleven translocation enzymes (Zhang X. et al., 2023; Mann et al., 2024), modulate histone decrotonylation and acylation, and provide acetyl groups for histone acetyltransferases (Xie et al., 2024).

Non-coding RNA networks further integrate microbial signals into host gene regulation. *H. pylori* upregulates miR-21 to target PTEN and PDCD4 (Behrouzi et al., 2020), while *Fusobacterium nucleatum* induces miR-155 via LPS/TLR4 signaling to suppress SOCS1 and promote immune evasion (Chen et al., 2022; Arre et al., 2024). Recent studies reveal that *Fusobacterium nucleatum*-derived extracellular vesicles deliver miR-1246 to target DAB2, disrupting the Hippo pathway (Wang P. et al., 2024). *H. pylori* also upregulates lncRNA H19, which sequesters let-7 to derepress HMGA2 and drive epithelial-mesenchymal transition (EMT) (Liu et al., 2024).

**Figure 2:**
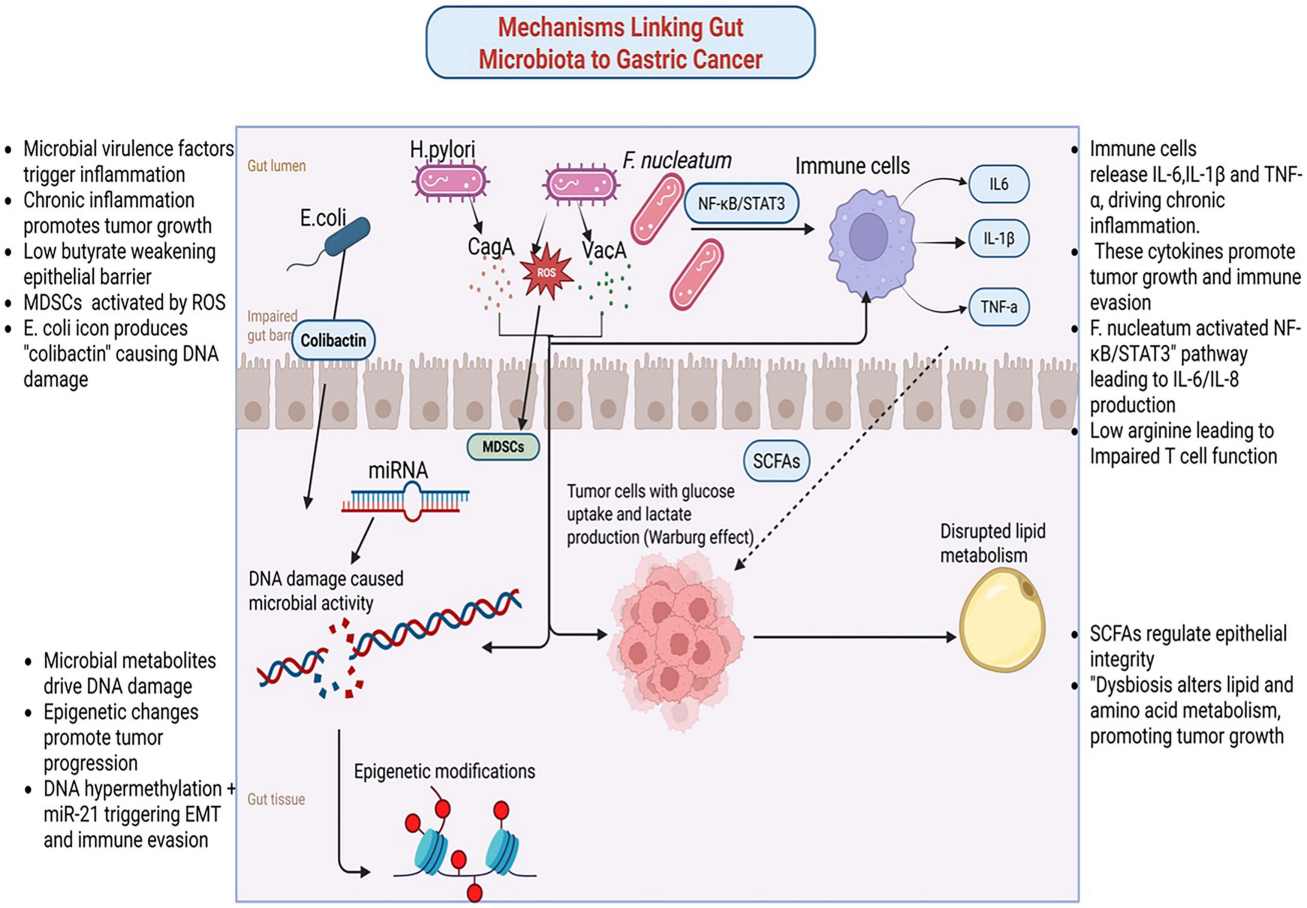
Mechanisms linking gut microbiome and gastric cancer. Created in Biorender.com.

Therapeutically, targeting this host-microbiota crosstalk shows significant promise. HDAC inhibitors (e.g., vorinostat) and DNMT inhibitors (e.g., azacytidine) reverse microbiota-driven epigenetic alterations and changes in gene expression, while phase I trials are evaluating anti-miR-21 oligonucleotides (Zhang et al., 2024). Additionally, CRISPR-engineered probiotics are being developed to restore protective butyrate production (Bianchetti et al., 2023). This mechanistic understanding of the epigenome-microbiome axis opens new avenues for preventing or reversing carcinogenic processes through precision targeting of microbial-epigenetic regulators (Figure 2; Table 2).

**Table 2:** Mechanisms linking gut microbiota to gastric cancer.

Aim of study	Main findings	Conclusions	References
To analyze the role of chronic inflammation in GC progression	*H. pylori* and *Fusobacterium nucleatum* activate NF-κB and STAT3 pathways, increasing inflammatory cytokine production and epithelial damage	Chronic inflammation induced by gut microbiota contributes to tumorigenesis	Chen et al. (2022)
To study the gut microbiota’s role in immunosuppression in GC	Dysbiosis suppresses anti-tumor immunity via Tregs and MDSCs Microbial metabolites like LPS contribute to immune evasion	Targeting gut microbiota-immunity interactions could enhance cancer immunotherapies	Dong et al. (2022)
To examine epigenetic and genetic modifications caused by microbiota	*H. pylori* and other microbes induce DNA methylation *H. pylori* and other microbes cause histone modifications and oncogenic microRNA expression	Microbial-driven epigenetic changes contribute to carcinogenesis and represent potential therapeutic targets	Jones and Neish (2017)
To investigate metabolic reprogramming mediated by gut microbiota	Microbial metabolites, such as SCFAs, ROS, and lactate, alter energy metabolism Promote immune evasion and tumor growth	Gut microbiota-induced metabolic changes influence GC progression and therapy resistance	Wu et al. (2024)
To assess how gut microbiota influences chemotherapy and immunotherapy efficacy	Dysbiosis can alter drug metabolism and reduce response rates to treatments like checkpoint inhibitors Some gut bacteria enhance chemotherapy effectiveness by modulating drug bioavailability	Understanding gut microbiota composition could lead to personalized cancer therapies	Guan et al. (2021)
To explore gut microbiota-derived toxins and their role in gastric carcinogenesis	Bacterial toxins, such as colibactin, hydrogen sulfide, and nitrosamines, promote DNA damage and mutations Increase oxidative stress, leading to genomic instability	Microbiota profiling could help identify high-risk patients for early GC screening	Cammarota et al. (2020)

## 5 Therapeutic modulations of gut microbiota in gastric cancer

### 5.1 Probiotics and prebiotics

GC is one of the most significant malignancies of the digestive system. The regulation of gut microbiota through probiotics and prebiotics represents a fundamental therapeutic strategy in GC management. The mechanism underlying their use in GC treatment include restoring microbial balance and exerting anti-inflammatory effects. Specific strains of *Lactobacillus* and *Bifidobacterium* exhibit probiotic properties that suppress *H. pylori*-induced gastric inflammation by enhancing mucosal immunity and reducing oxidative stress (Nabavi-Rad et al., 2022). These strains promote the production of anti-inflammatory cytokines, such as IL-10 and TGF-β, while inhibiting pro-inflammatory cytokines, including TNF-α. Additionally, probiotics strengthen the gastric mucosal barrier by increasing mucin secretion and preserving epithelial integrity, thereby preventing microbial translocation and systemic inflammation (He et al., 2023).

Prebiotics, including inulin, fructooligosaccharides (FOS), and galactooligosaccharides (GOS), serve as substrates for beneficial gut bacteria, stimulating the production of SCFAs, including butyrate, acetate, and propionate. For example, butyrate acts as a HDAC inhibitor, enhancing anti-inflammatory and anti-carcinogenic gene expression in gastric tissues (Salek Farrokhi et al., 2020). Moreover, human trials have revealed that dietary interventions with prebiotics also improve the effectiveness of conventional therapies, including chemotherapy and immunotherapy, through modulation of the gut microbiota composition and improving treatment outcomes (Yao et al., 2025).

### 5.2 Antibiotics and *Helicobacter pylori* eradication

Antibiotics play a crucial role in treating GC associated with *H. pylori* (Piscione et al., 2021). In high-risk individuals, early eradication of *H. pylori* has been shown to significantly reduce the likelihood of developing GC. However, the use of antibiotics is not without risks. Extensive patient exposure and inappropriate use by uninformed individuals have led to overuse and misuse, resulting in antibiotic resistance, which poses a significant challenge to the effectiveness of *H. pylori* eradication therapies. Additionally, broad-spectrum antibiotics can disrupt the gut microbiota, leading to dysbiosis, reduced microbial diversity, and impaired immune responses (Fishbein et al., 2023). To address these limitations, adjunctive probiotic supplementation and the development of novel anti-microbial agents are being explored to enhance the efficacy and safety of antibiotic therapy in GC management.

### 5.3 Dietary interventions

Given the potential role of dysbiosis in GC development, understanding how to promote a healthy gut environment is essential, particularly in the context of gastric tumorigenesis. Dietary interventions are non-invasive, sustainable tools for modulating microbiota and lowering risk for GC. Specific dietary patterns influence the composition and function of the gut microbiota, with certain diets associated with favorable microbial profiles (Rinninella et al., 2019). A diet rich in fiber, whole grains, fruits, and vegetables promotes the growth of beneficial gut microbiota and the synthesis of SCFAs, particularly butyrate, which exhibits anti-inflammatory and anti-carcinogenic properties (Bultman, 2017). High-fiber diets are also linked to enhanced immune function, decreased oxidative stress, and a strengthened gut barrier, all of which reduce the risk of GC.

Polyphenols, bioactive compounds found in foods such as berries, green tea, and dark chocolate, further contribute to gut microbiota modulation (Plamada and Vodnar, 2021). These compounds act as fermentation substrates for beneficial bacterial like *Bifidobacterium* and *Lactobacillus*. In gastric tissues, polyphenol-derived metabolites have been shown to induce apoptosis and inhibit angiogenesis, thereby suppressing tumor growth (Gade and Kumar, 2023). Additionally, plant-based diets are rich in antioxidants and phytochemicals that counteract oxidative stress and inflammation, key drivers of GC (Guan et al., 2021). While dietary interventions hold promise, individual variability in gut microbiota composition and dietary responses highlights the potential need for personalized nutrition strategies to maximize their therapeutic potential.

### 5.4 Fecal microbiota transplantation

**Figure 3:**
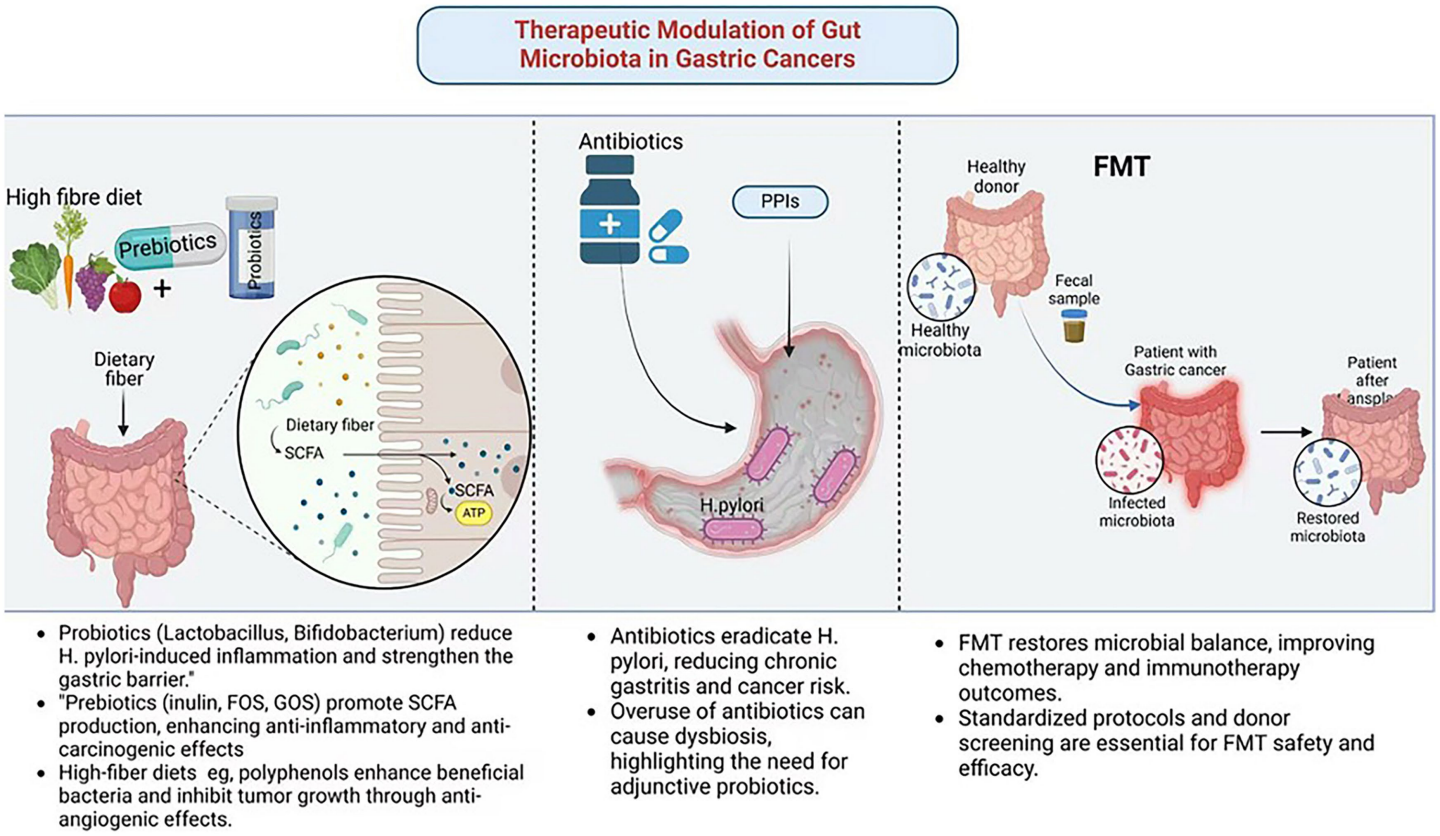
Therapeutic modulation of gut microbiota in gastric cancer. Created in Biorender.com.

FMT, a novel therapy involving the transfer of feces from a healthy donor to a recipient, offers the potential to restore a balanced gut microbiota (Biazzo and Deidda, 2022). FMT has gained attention as a potential treatment for dysbiosis-related diseases, including GC. Preclinical and clinical studies have demonstrated that FMT can alter gut microbiota, with reports in patients with GC showing increased microbial diversity and the growth of beneficial bacteria. These changes have been associated with improved efficacy of chemotherapy and immunotherapy (Park et al., 2020). FMT has also been shown to reduce systemic inflammation and restore immune responses, thereby curbing tumor progression (Tian et al., 2024) (Figure 3; Table 3).

**Table 3:** Therapeutic modulation of gut microbiota in gastric cancer.

Aim of study	Main findings	Conclusions	References
To evaluate the efficacy of probiotics and prebiotics for GC	Probiotics, such as *Lactobacillus* and *Bifidobacterium*, reduce inflammation and enhance gut barrier integrity Probiotics modulate immune responses	Probiotics and prebiotics are promising interventions but require strain-specific optimization and further clinical validation	He et al. (2023)
To assess the impact of antibiotics and *H. pylori* eradication	Eradication of *H. pylori* reduces gastric inflammation and cancer risk Potential risks include microbiota disruption and antibiotic resistance	Antibiotic use must be carefully balanced with strategies to preserve microbiota integrity to avoid unintended dysbiosis	Piscione et al. (2021)
To explore dietary interventions in modulating gut microbiota	High-fiber and polyphenol-rich diets promote beneficial bacteria and SCFAs production Reduce oxidative stress and inflammation	Dietary modifications are a key strategy for maintaining gut homeostasis and may contribute to GC prevention	Bultman (2017) and Rinninella et al. (2019)
To examine the potential of FMT in GC treatment	FMT restores microbial diversity Improves chemotherapy response Reduces systemic inflammation	FMT is a promising therapeutic approach but remains experimental, requiring further clinical trials and standardized safety protocols	Park et al. (2020)
To assess microbiota-derived biomarkers for early GC detection	Specific microbial signatures correlate with early-stage GC Non-invasive stool and saliva microbiome tests show promise for diagnostics	Microbiota-based biomarkers could enhance early detection and risk stratification	Liu et al. (2024) and Mukherjee et al. (2025)
To evaluate the long-term safety and feasibility of microbiota-targeted therapies	Probiotic-based therapies are generally safe but have variable efficacy Long-term impacts of microbiota alteration remain unclear	More controlled studies are needed to establish safety guidelines for clinical applications	Yue et al. (2021)

## 6 Diagnostic and prognostic implications of gut microbiota

### 6.1 Microbial biomarkers for early detection

The gut microbiota harbors microbial biomarkers that offer significant diagnostic potential for GC. Research has shown that the composition of the gut microbiota varies among individuals with different stages of gastric disease, including superficial gastritis, atrophic gastritis, gastric mucosal atypical hyperplasia, and advanced GC, distinct microbial compositional changes were identified (Miao et al., 2022). For instance, dysbiosis in GC is often characterized by an overabundance of pro-inflammatory bacterial species, such as *Fusobacterium nucleatum* and *Escherichia coli*, alongside a reduction in beneficial microbes like *Lactobacillus* and *Bifidobacterium* (Mukherjee et al., 2025). Non-invasive methods, such as stool and saliva microbiota analysis, have emerged as promising tools for early GC detection. Another study demonstrated that a combination of *Lactobacillus* and *Streptococcus* in fecal samples could effectively discriminate between GC patients and healthy individuals, with an area under the curve (AUC) of 0.7949 in one analysis and 0.7712 in an independent cohort, suggesting their potential as non-invasive diagnostic markers (Wang Y. et al., 2024).

### 6.2 Predictive models for treatment outcomes

**Figure 4:**
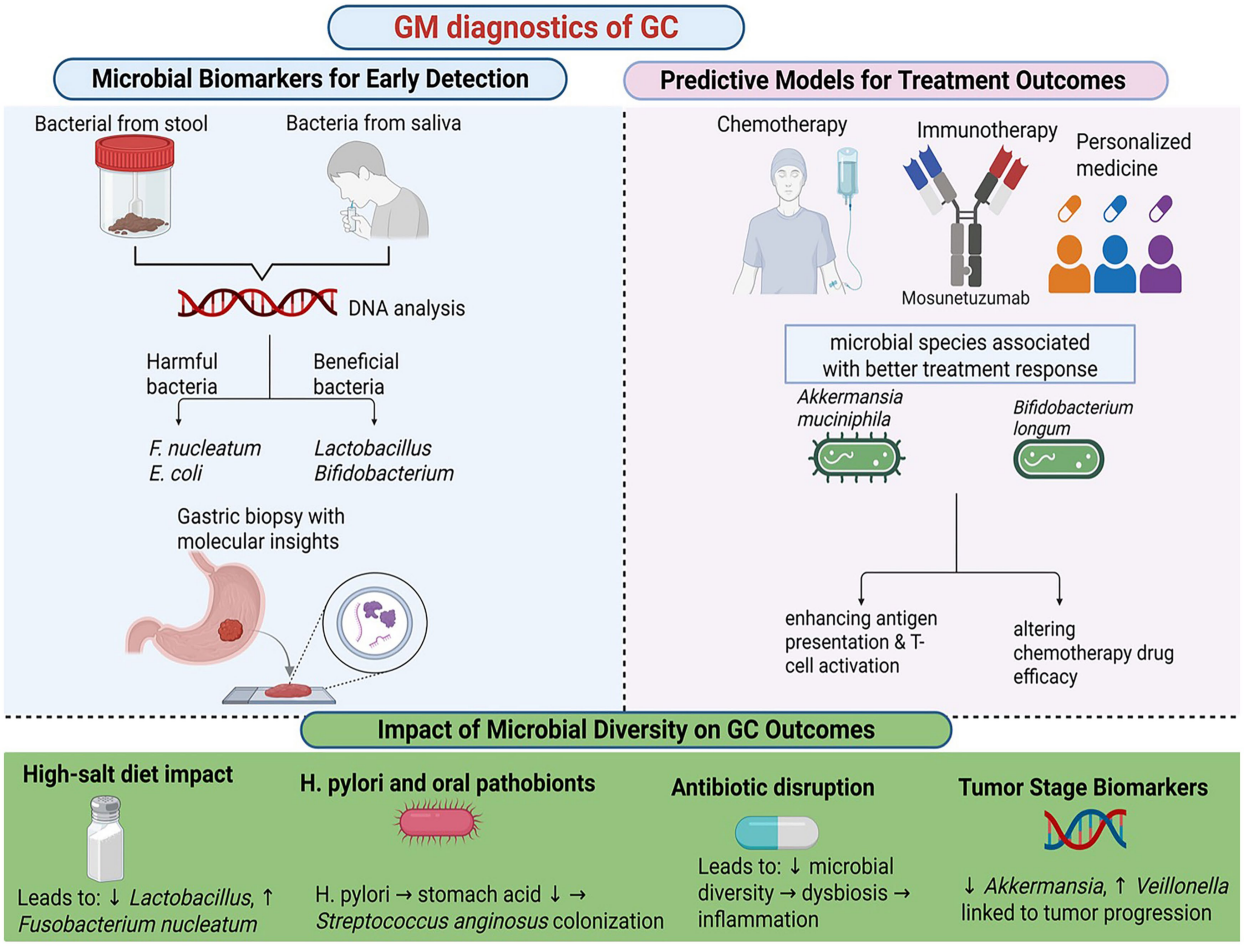
Gut microbiota diagnostic of gastric cancer. Created in Biorender.com.

The gut microbiota significantly influences the efficacy of GC treatments, including chemotherapy, immunotherapy, and precision medicine. Emerging evidence suggests that microbiota-based predictors can stratify patients and guide personalized therapeutic strategies. For example, the response of immunotherapy patients to immune checkpoint inhibitors has been shown to correlate with gut microbiota composition (Tomela et al., 2020). Specific microbial species, such as *Akkermansia muciniphila* and *Bifidobacterium longum*, have been associated with immune checkpoint inhibitors in patients with GC (Kiousi et al., 2023). These microbes not only enhance antigen presentation and T cell activation, thereby boosting anti-tumor immunity, but also modulate the tumor microenvironment. The gut microbiota also plays a role in chemotherapy outcomes by interacting with chemotherapeutic drugs. Certain bacteria produce enzymes that metabolize these drugs, altering their efficacy and toxicity. For instance, microbial metabolism of irinotecan generates toxic metabolites that exacerbate gastrointestinal side effects (Yue et al., 2021). Conversely, microbiota profiles that promote the production of SCFAs have been linked to reduced toxicity and better treatment tolerance (Figure 4).

## 7 Challenges and future directions

### 7.1 Limitations of existing microbiota-targeted therapies

Microbiota-targeted therapies, including probiotics, prebiotics, and FMT, have shown promise in clinical applications. However, several limitations hinder their broader adoption. One major limitation is the lack of standardized protocols. Variability in probiotic strains, dosages, and delivery methods complicates the generalization of findings across studies and populations. For example, while FMT has proven effective in treating recurrent *Clostridium Difficile* infections, its application in cancer patients remains limited due to logistical and safety concerns, such as the risk of transmitting infections and introducing pathogenic microbes (Sandhu and Chopra, 2021). Additionally, the long-term effects of altering gut microbial communities are poorly understood, raising concerns about potential unintended consequences. The inconsistent efficacy of microbiota-targeted therapies in GC further underscores the need for personalized approaches that consider individual microbiota profiles and genetic predispositions (Nagpal et al., 2018).

### 7.2 Challenges in clinical translation

One of the most significant challenges in microbiota research is the variability of gut microbiota across individuals. Factors such as diet, age, genetics, and geographical location contribute to this variability, making it difficult to develop universal microbiota-based therapies (Sandoval-Motta et al., 2017). Research has shown that gut microbiota composition varies widely between populations from different geographical regions, highlighting the influence of local environmental conditions, dietary habits, and nutritional intake on microbial diversity (Gupta et al., 2017; Healey et al., 2017). In addition, research using germ-free mice humanized with microbiome samples from donors of different countries have demonstrated that geographic origin can affect susceptibility to enteric infections like *Citrobacter rodentium* (Porras et al., 2021). While host genetics play a role in microbiome variability, environmental factors such as shared household environments often exert a more pronounced effect. To address this, future research should focus on personalized microbiota interventions, leveraging multi-omics technologies and machine learning to tailor therapies based on individual microbiota profiles.

Another critical issue is the standardization of FMT protocols. The absence of standardized protocols for FMT represents a significant obstacle to its broad clinical adoption. Heterogeneity in donor selection, fecal processing methodologies, and administration techniques complicates the interpretation of research outcomes and restricts the generalizability of findings. Recent efforts have been made to address these challenges, such as the joint workshop by the International Alliance for Biological Standardization (IABS) and the BIOASTER Microbiology Technology Institute, which aimed to provide a multidisciplinary perspective on developing FMT guidelines, including technical, regulatory, and standardization requirements (Servetas et al., 2022). Variations in fecal processing approaches, such as the use of fresh versus frozen samples, may influence the viability and composition of the transplanted microbiota. Furthermore, concerns persist regarding the safety of FMT in cancer patients, given the incomplete understanding of risks associated with introducing pathogenic microbes or disturbing the recipient’s microbiota. To ensure the safe and effective utilization of FMT in GC management, it is imperative to establish comprehensive standardized guidelines encompassing stringent donor screening and robust quality control measures (Karimi et al., 2024).

Ethical and logistical considerations also play a significant role in microbiota-based interventions. Ensuring patient safety is paramount, particularly in vulnerable populations like cancer patients. For example, the potential risks associated with FMT, including the transmission of infections and unintended alterations to the recipient’s microbiota, must be carefully managed (Merrick et al., 2020). Ethical considerations also extend to the use of microbiota data, as issues of data privacy and informed consent become increasingly relevant (Ma et al., 2018). Patients must be adequately informed about how their microbiota data will be used and protected. Logistically, the scalability of microbiota-based therapies is a significant challenge, as the infrastructure and economic investment required for large-scale production and distribution are substantial (Rabaey et al., 2020). Addressing these challenges will require collaboration among researchers, clinicians, and policymakers to develop ethical guidelines and scalable solutions.

### 7.3 Inter-study comparability issues

Inter-study comparability is a significant challenge in microbiota research, primarily due to variability in experimental design, heterogeneity in study populations, and methodological differences. Differences in experimental design, such as variations in sample size, follow-up duration, and control group selection, can lead to inconsistent results. Studies with small sample sizes may lack the statistical power to detect significant effects, while those with short follow-up periods may fail to capture the long-term impacts of microbiota interventions. To enhance comparability, future research should adopt standardized experimental designs, including predefined sample size calculations and follow-up protocols.

The heterogeneity of study populations further complicates the interpretation of microbiota research. Differences in age, gender, disease stage, and geographical location can influence microbiota composition and intervention outcomes. For instance, elderly patients often exhibit reduced microbiota diversity compared to younger individuals, which may affect their response to probiotics or prebiotics. Similarly, dietary habits and environmental exposures vary across regions, further contributing to population-specific differences. To address this, future studies should aim to include diverse and representative cohorts, enabling a more comprehensive understanding of microbiota dynamics in GC.

Variability in microbiota analysis techniques and data processing methods also hinders inter-study comparability. For example, 16S rRNA sequencing and metagenomic sequencing differ in their resolution and coverage, leading to potential discrepancies in the identification of microbial taxa. Additionally, differences in bioinformatics pipelines, such as the use of operational taxonomic units (OTUs) versus amplicon sequence variants (ASVs), can affect the interpretation of results. To minimize these discrepancies, researchers should adopt standardized protocols for microbiota analysis and data processing, and transparently report methodological details in their publications.

### 7.4 Advances in gut microbiota research

Recent developments in gut microbiome research have opened promising avenues for overcoming these challenges. The use of multi-omics methods, such as metagenomics, transcriptomic, proteomics, and metabolomics, has revolutionized the study of microbiota-host interactions (Sanches et al., 2024). These methods enable researchers to examine microbial communities in depth and determine their role in health and disease. Significant progress has also been made in personalized medicine, allowing researchers to develop tailored therapeutic strategies based on individual microbiota profiles by integrating clinical and environmental factors with machine learning and multi-omics data. For example, personalized probiotics and prebiotics are being studied to optimize gut health and improve treatment outcomes in GC patients (Bianchetti et al., 2023). Moreover, precision nutrition, an emerging strategy that uses microbiota data to tailor dietary interventions, has advanced as a complementary approach for cancer management (Shukla et al., 2024).

Artificial intelligence (AI) and machine learning are also transforming microbiota research. These technologies enable the analysis of large datasets to identify microbial biomarkers, predict response to treatment, and uncover novel therapy targets. Integrating microbiota data with other omics layers through AI-driven algorithms has broadened the scope of research, leading to more accurate diagnostics and targeted therapies (Biswas and Chakrabarti, 2020). Collaborative efforts, such as the ImmUniverse Consortium, are further advancing personalized medicine by combining multi-omics approaches combined with clinical data to build predictive models for improved decision-making and patient to outcomes in immune-mediated diseases like GC.

### 7.5 Future directions

**Figure 5:**
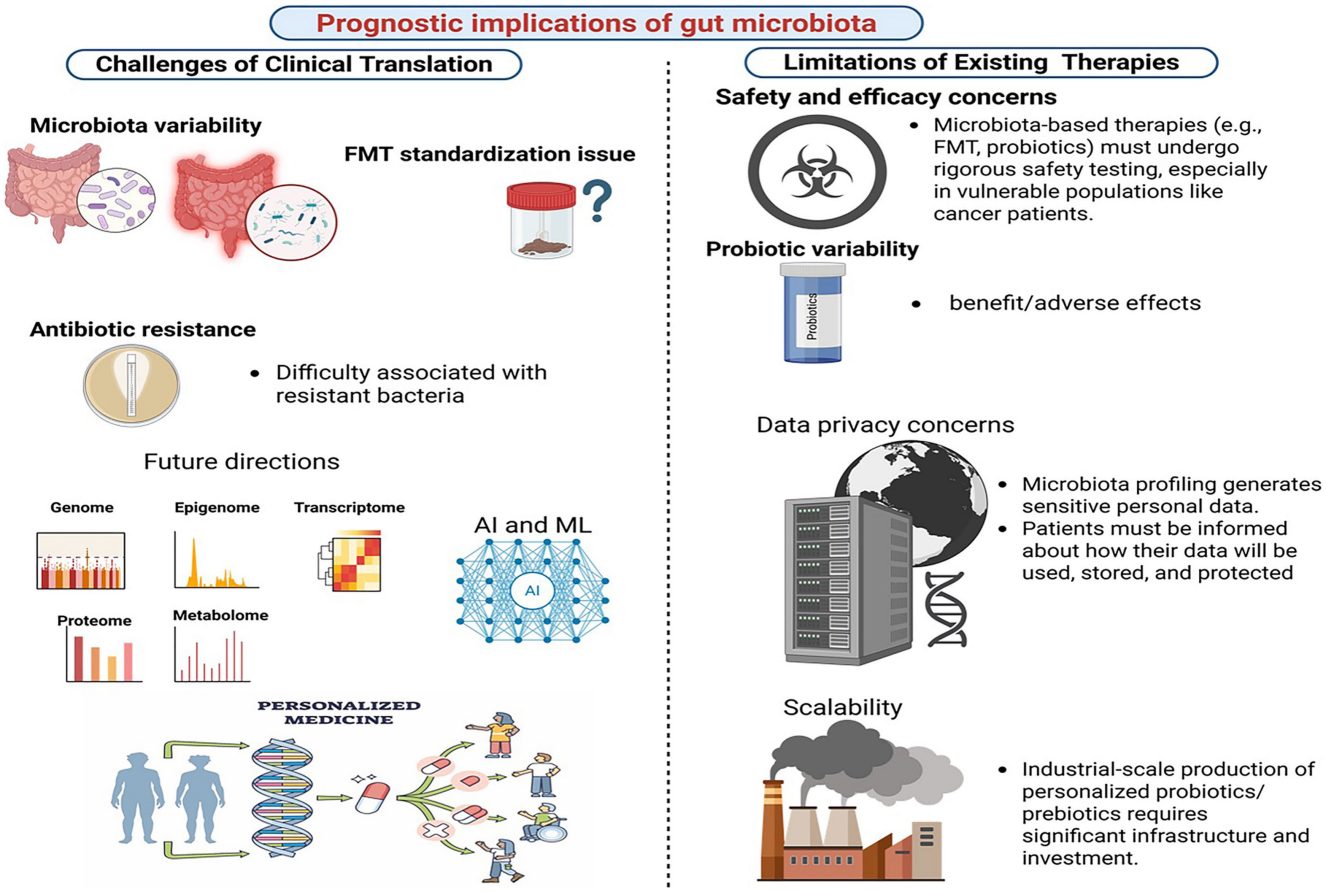
Prognostics implications of gut microbiome for gastric cancer. Created in Biorender.com.

To address current limitations, we propose key future directions for microbiota research. First, standardized protocols for microbiota analysis and intervention delivery are needed to enhance reproducibility and comparability. Second, personalized microbiota interventions, such as probiotics, prebiotics, and precision nutrition, should be expanded to optimize therapeutic outcomes across diverse populations. Third, robust computational tools are essential for integrating and interpreting multi-omics data, enabling a deeper understanding of microbiota-host interactions. Fourth, AI-driven models should be validated in independent cohorts to improve their utility in predicting treatment responses and identifying therapeutic targets. Finally, international collaborations and multi-center studies should be fostered to harmonize protocols, share data, and improve the generalizability of findings. These efforts will accelerate the translation of microbiota-based therapies into clinical practice (Figure 5).

## 8 Conclusion

The study of gut microbiota in GC has unveiled its critical role in tumorigenesis, progression, and therapeutic outcomes. Dysbiosis drives chronic inflammation, immune modulation, metabolic reprogramming, and epigenetic changes, contributing to gastric carcinogenesis. Key microbes like *H. pylori* and microbial metabolites exhibit context-dependent roles in tumor growth, highlighting the complexity of microbiota-host interactions. Therapeutic strategies, including probiotics, prebiotics, dietary interventions, antibiotics, and FMT, offer promise in restoring microbial balance, enhancing treatment efficacy, and reducing toxicity. Advances in microbial biomarkers and multi-omics technologies further enable early diagnosis and personalized medicine. Integrating microbiota modulation into clinical practice represents a transformative opportunity to shift from reactive treatment to proactive prevention, paving the way for predictive, preventive, and personalized care in the management of GC.

## References

[ref1] Alvarez J. Fernandez Real J. M. Guarner F. Gueimonde M. Rodriguez J. M. Saenz de Pipaon M.. (2021). Gut microbes and health. Gastroenterol. Hepatol. 44, 519–535. doi: 10.1016/j.gastrohep.2021.01.009, PMID: 33652061

[ref2] Arre V. Mastrogiacomo R. Balestra F. Serino G. Viti F. Rizzi F.. (2024). Unveiling the potential of extracellular vesicles as biomarkers and therapeutic Nanotools for gastrointestinal diseases. Pharmaceutics 16:567. doi: 10.3390/pharmaceutics16040567, PMID: 38675228 PMC11055174

[ref3] Behrouzi A. Ashrafian F. Mazaheri H. Lari A. Nouri M. Riazi Rad F.. (2020). The importance of interaction between MicroRNAs and gut microbiota in several pathways. Microb. Pathog. 144:104200. doi: 10.1016/j.micpath.2020.104200, PMID: 32289465

[ref4] Bemark M. Pitcher M. J. Dionisi C. Spencer J. (2024). Gut-associated lymphoid tissue: a microbiota-driven hub of B cell immunity. Trends Immunol. 45, 211–223. doi: 10.1016/j.it.2024.01.006, PMID: 38402045 PMC11227984

[ref5] Bhattacharjee A. Sahoo O. S. Sarkar A. Bhattacharya S. Chowdhury R. Kar S.. (2024). Infiltration to infection: key virulence players of *Helicobacter pylori* pathogenicity. Infection 52, 345–384. doi: 10.1007/s15010-023-02159-9, PMID: 38270780

[ref6] Bianchetti G. De Maio F. Abeltino A. Serantoni C. Riente A. Santarelli G.. (2023). Unraveling the gut microbiome-diet connection: exploring the impact of digital precision and personalized nutrition on microbiota composition and host physiology. Nutrients 15:3931. doi: 10.3390/nu15183931, PMID: 37764715 PMC10537332

[ref7] Biazzo M. Deidda G. (2022). Fecal microbiota transplantation as new therapeutic avenue for human diseases. J. Clin. Med. 11:4119. doi: 10.3390/jcm11144119, PMID: 35887883 PMC9320118

[ref8] Biswas N. Chakrabarti S. (2020). Artificial intelligence (AI)-based systems biology approaches in multi-omics data analysis of Cancer. Front. Oncol. 10:588221. doi: 10.3389/fonc.2020.588221, PMID: 33154949 PMC7591760

[ref9] Bossuet-Greif N. Vignard J. Taieb F. Mirey G. Dubois D. Petit C.. (2018). The colibactin genotoxin generates DNA interstrand cross-links in infected cells. MBio 9:e02393-17. doi: 10.1128/mBio.02393-17, PMID: 29559578 PMC5874909

[ref10] Bultman S. J. (2017). Interplay between diet, gut microbiota, epigenetic events, and colorectal cancer. Mol. Nutr. Food Res. 61, 1–12. doi: 10.1002/mnfr.201500902, PMID: 27138454 PMC5161716

[ref11] Cammarota G. Ianiro G. Ahern A. Carbone C. Temko A. Claesson M. J.. (2020). Gut microbiome, big data and machine learning to promote precision medicine for cancer. Nat. Rev. Gastroenterol. Hepatol. 17, 635–648. doi: 10.1038/s41575-020-0327-3, PMID: 32647386

[ref12] Casalegno Garduno R. Dabritz J. (2021). New insights on CD8(+) T cells in inflammatory bowel disease and therapeutic approaches. Front. Immunol. 12:738762. doi: 10.3389/fimmu.2021.738762, PMID: 34707610 PMC8542854

[ref13] Cebra J. J. Periwal S. B. Lee G. Lee F. Shroff K. E. (1998). Development and maintenance of the gut-associated lymphoid tissue (GALT): the roles of enteric bacteria and viruses. Dev. Immunol. 6, 13–18. doi: 10.1155/1998/68382, PMID: 9716901 PMC2276005

[ref14] Chen Y. Huang Z. Tang Z. Huang Y. Huang M. Liu H.. (2022). More than just a periodontal pathogen -the research Progress on *Fusobacterium nucleatum*. Front. Cell. Infect. Microbiol. 12:815318. doi: 10.3389/fcimb.2022.815318, PMID: 35186795 PMC8851061

[ref15] Cheng Y. He C. Wang M. Ma X. Mo F. Yang S.. (2019). Targeting epigenetic regulators for cancer therapy: mechanisms and advances in clinical trials. Signal Transduct. Target. Ther. 4:62. doi: 10.1038/s41392-019-0095-0, PMID: 31871779 PMC6915746

[ref16] Choi Y. Bose S. Seo J. Shin J. H. Lee D. Kim Y.. (2021). Effects of live and pasteurized forms of Akkermansia from the human gut on obesity and metabolic dysregulation. Microorganisms 9:2039. doi: 10.3390/microorganisms9102039, PMID: 34683361 PMC8538271

[ref17] Conti L. Borro M. Milani C. Simmaco M. Esposito G. Canali G.. (2021). Gastric microbiota composition in patients with corpus atrophic gastritis. Dig. Liver Dis. 53, 1580–1587. doi: 10.1016/j.dld.2021.05.005, PMID: 34116969

[ref18] Dadarwal D. Palmer C. Griebel P. (2017). Mucosal immunity of the postpartum bovine genital tract. Theriogenology 104, 62–71. doi: 10.1016/j.theriogenology.2017.08.010, PMID: 28822242

[ref19] Demirkiran N. Aydin B. Pehlivan M. Yuce Z. Sercan H. O. (2024). Study of the effect of sFRP1 protein on molecules involved in the regulation of DNA methylation in CML cell line. Med. Oncol. 41:109. doi: 10.1007/s12032-024-02336-2, PMID: 38592567

[ref20] Ding S. Z. Goldberg J. B. Hatakeyama M. (2010). *Helicobacter pylori* infection, oncogenic pathways and epigenetic mechanisms in gastric carcinogenesis. Future Oncol. 6, 851–862. doi: 10.2217/fon.10.37, PMID: 20465395 PMC2882595

[ref21] Dong Y. Yang Q. Niu R. Zhang Z. Huang Y. Bi Y.. (2022). Modulation of tumor-associated macrophages in colitis-associated colorectal cancer. J. Cell. Physiol. 237, 4443–4459. doi: 10.1002/jcp.30906, PMID: 36302153

[ref22] Fan X. Jin J. Yan L. Liu L. Li Q. Xu Y. (2020). The impaired anti-tumoral effect of immune surveillance cells in the immune microenvironment of gastric cancer. Clin. Immunol. 219:108551. doi: 10.1016/j.clim.2020.108551, PMID: 32739413

[ref23] Feng P. Xiao X. Zhou T. Li X. (2020). “Effects of the bio-accumulative environmental pollutants on the gut microbiota” in Gut remediation of environmental pollutants: Potential roles of probiotics and gut microbiota. eds. Li X. Liu P. (Singapore: Springer Singapore), 109–143.

[ref24] Fishbein S. R. S. Mahmud B. Dantas G. (2023). Antibiotic perturbations to the gut microbiome. Nat. Rev. Microbiol. 21, 772–788. doi: 10.1038/s41579-023-00933-y, PMID: 37491458 PMC12087466

[ref25] Gade A. Kumar M. S. (2023). Gut microbial metabolites of dietary polyphenols and their potential role in human health and diseases. J. Physiol. Biochem. 79, 695–718. doi: 10.1007/s13105-023-00981-1, PMID: 37653220

[ref26] Ghaffari S. Abbasi A. Somi M. H. Moaddab S. Y. Nikniaz L. Kafil H. S.. (2023). *Akkermansia muciniphila*: from its critical role in human health to strategies for promoting its abundance in human gut microbiome. Crit. Rev. Food Sci. Nutr. 63, 7357–7377. doi: 10.1080/10408398.2022.2045894, PMID: 35238258

[ref27] Gill P. A. van Zelm M. C. Muir J. G. Gibson P. R. (2018). Review article: short chain fatty acids as potential therapeutic agents in human gastrointestinal and inflammatory disorders. Aliment. Pharmacol. Ther. 48, 15–34. doi: 10.1111/apt.14689, PMID: 29722430

[ref28] Guan R. Van Le Q. Yang H. Zhang D. Gu H. Yang Y.. (2021). A review of dietary phytochemicals and their relation to oxidative stress and human diseases. Chemosphere 271:129499. doi: 10.1016/j.chemosphere.2020.129499, PMID: 33445014

[ref29] Gupta V. K. Paul S. Dutta C. (2017). Geography, ethnicity or subsistence-specific variations in human microbiome composition and diversity. Front. Microbiol. 8:1162. doi: 10.3389/fmicb.2017.01162, PMID: 28690602 PMC5481955

[ref30] Hahm J. Y. Park J. Jang E.-S. Chi S. W. (2022). 8-Oxoguanine: from oxidative damage to epigenetic and epitranscriptional modification. Exp. Mol. Med. 54, 1626–1642. doi: 10.1038/s12276-022-00822-z, PMID: 36266447 PMC9636213

[ref31] Han L. Shu X. Wang J. (2022). *Helicobacter pylori*-mediated oxidative stress and gastric diseases: a review. Front. Microbiol. 13:811258. doi: 10.3389/fmicb.2022.811258, PMID: 35211104 PMC8860906

[ref32] He C. Gao H. Xin S. Hua R. Guo X. Han Y.. (2023). View from the biological property: insight into the functional diversity and complexity of the gut mucus. Int. J. Mol. Sci. 24:4227. doi: 10.3390/ijms24044227, PMID: 36835646 PMC9960128

[ref33] Healey G. R. Murphy R. Brough L. Butts C. A. Coad J. (2017). Interindividual variability in gut microbiota and host response to dietary interventions. Nutr. Rev. 75, 1059–1080. doi: 10.1093/nutrit/nux062, PMID: 29190368

[ref34] Jia W. Xie G. Jia W. (2018). Bile acid-microbiota crosstalk in gastrointestinal inflammation and carcinogenesis. Nat. Rev. Gastroenterol. Hepatol. 15, 111–128. doi: 10.1038/nrgastro.2017.119, PMID: 29018272 PMC5899973

[ref35] Jones R. M. Neish A. S. (2017). Redox signaling mediated by the gut microbiota. Free Radic. Biol. Med. 105, 41–47. doi: 10.1016/j.freeradbiomed.2016.10.495, PMID: 27989756

[ref36] Karimi M. Shirsalimi N. Hashempour Z. Salehi Omran H. Sedighi E. Beigi F.. (2024). Safety and efficacy of fecal microbiota transplantation (FMT) as a modern adjuvant therapy in various diseases and disorders: a comprehensive literature review. Front. Immunol. 15:1439176. doi: 10.3389/fimmu.2024.1439176, PMID: 39391303 PMC11464302

[ref37] Keikha M. Karbalaei M. (2021). Probiotics as the live microscopic fighters against *Helicobacter pylori* gastric infections. BMC Gastroenterol. 21:388. doi: 10.1186/s12876-021-01977-1, PMID: 34670526 PMC8527827

[ref38] Kiousi D. E. Kouroutzidou A. Z. Neanidis K. Karavanis E. Matthaios D. Pappa A.. (2023). The role of the gut microbiome in Cancer immunotherapy: current knowledge and future directions. Cancers (Basel) 15:2101. doi: 10.3390/cancers15072101, PMID: 37046762 PMC10093606

[ref39] Kontizas E. Tastsoglou S. Karamitros T. Karayiannis Y. Kollia P. Hatzigeorgiou A. G.. (2020). Impact of *Helicobacter pylori* infection and its major virulence factor CagA on DNA damage repair. Microorganisms 8:2007. doi: 10.3390/microorganisms8122007, PMID: 33339161 PMC7765595

[ref40] La Rosa G. R. M. Gattuso G. Pedulla E. Rapisarda E. Nicolosi D. Salmeri M. (2020). Association of oral dysbiosis with oral cancer development. Oncol. Lett. 19, 3045–3058. doi: 10.3892/ol.2020.11441, PMID: 32211076 PMC7079586

[ref41] Lahner E. Conti L. Annibale B. Corleto V. D. (2020). Current perspectives in atrophic gastritis. Curr. Gastroenterol. Rep. 22:38. doi: 10.1007/s11894-020-00775-1, PMID: 32542467

[ref42] Larsen J. M. (2017). The immune response to Prevotella bacteria in chronic inflammatory disease. Immunology 151, 363–374. doi: 10.1111/imm.12760, PMID: 28542929 PMC5506432

[ref43] Le Noci V. Bernardo G. Bianchi F. Tagliabue E. Sommariva M. Sfondrini L. (2021). Toll like receptors as sensors of the tumor microbial Dysbiosis: implications in Cancer progression. Front. Cell Dev. Biol. 9:732192. doi: 10.3389/fcell.2021.732192, PMID: 34604233 PMC8485072

[ref44] Li J. Cai H. Zhang Y. Li J. Wang D. Li H.. (2024). Dysbiosis of gut microbiota is associated with pathogenesis of peptic ulcer diseases through inflammatory proteins: a Mendelian randomization study. Medicine (Baltimore) 103:e39814. doi: 10.1097/MD.0000000000039814, PMID: 39331926 PMC11441939

[ref45] Liu K. Yang X. Zeng M. Yuan Y. Sun J. He P.. (2021). The role of fecal fusobacterium nucleatum and pks(+) *Escherichia coli* as early diagnostic markers of colorectal Cancer. Dis. Markers 2021:1171239. doi: 10.1155/2021/1171239, PMID: 34853619 PMC8629656

[ref46] Liu Z. Zhang D. Chen S. (2024). Unveiling the gastric microbiota: implications for gastric carcinogenesis, immune responses, and clinical prospects. J. Exp. Clin. Cancer Res. 43:118. doi: 10.1186/s13046-024-03034-7, PMID: 38641815 PMC11027554

[ref47] Luu M. Schutz B. Lauth M. Visekruna A. (2023). The impact of gut microbiota-derived metabolites on the tumor immune microenvironment. Cancers (Basel) 15:1588. doi: 10.3390/cancers15051588, PMID: 36900377 PMC10001145

[ref48] Ma Y. Chen H. Lan C. Ren J. (2018). Help, hope and hype: ethical considerations of human microbiome research and applications. Protein Cell 9, 404–415. doi: 10.1007/s13238-018-0537-4, PMID: 29675808 PMC5960465

[ref49] Ma J. Piao X. Mahfuz S. Long S. Wang J. (2022). The interaction among gut microbes, the intestinal barrier and short chain fatty acids. Animal Nutrition 9, 159–174. doi: 10.1016/j.aninu.2021.09.012, PMID: 35573092 PMC9079705

[ref50] Malesza I. J. Malesza M. Walkowiak J. Mussin N. Walkowiak D. Aringazina R.. (2021). High-fat, western-style diet, systemic inflammation, and gut microbiota: a narrative review. Cells 10:3164. doi: 10.3390/cells10113164, PMID: 34831387 PMC8619527

[ref51] Mann E. R. Lam Y. K. Uhlig H. H. (2024). Short-chain fatty acids: linking diet, the microbiome and immunity. Nat. Rev. Immunol. 24, 577–595. doi: 10.1038/s41577-024-01014-8, PMID: 38565643

[ref52] Merrick B. Allen L. Masirah M. Z. N. Forbes B. Shawcross D. L. Goldenberg S. D. (2020). Regulation, risk and safety of faecal microbiota transplant. Infect. Prev. Pract. 2:100069. doi: 10.1016/j.infpip.2020.100069 34316559 PMC7280140

[ref53] Mestre L. Carrillo-Salinas F. J. Mecha M. Feliu A. Guaza C. (2018). Gut microbiota, cannabinoid system and neuroimmune interactions: new perspectives in multiple sclerosis. Biochem. Pharmacol. 157, 51–66. doi: 10.1016/j.bcp.2018.08.037, PMID: 30171835

[ref54] Miao Y. Tang H. Zhai Q. Liu L. Xia L. Wu W.. (2022). Gut microbiota Dysbiosis in the development and progression of gastric Cancer. J. Oncol. 2022, 9971619–9971615. doi: 10.1155/2022/9971619, PMID: 36072968 PMC9441395

[ref55] Miller P. L. Carson T. L. (2020). Mechanisms and microbial influences on CTLA-4 and PD-1-based immunotherapy in the treatment of cancer: a narrative review. Gut Pathog 12:43. doi: 10.1186/s13099-020-00381-6, PMID: 32944086 PMC7488430

[ref56] Mima K. Ogino S. Nakagawa S. Sawayama H. Kinoshita K. Krashima R.. (2017). The role of intestinal bacteria in the development and progression of gastrointestinal tract neoplasms. Surg. Oncol. 26, 368–376. doi: 10.1016/j.suronc.2017.07.011, PMID: 29113654 PMC5726560

[ref57] Mostafavi Abdolmaleky H. Zhou J. R. (2024). Gut microbiota Dysbiosis, oxidative stress, inflammation, and epigenetic alterations in metabolic diseases. Antioxidants (Basel) 13:985. doi: 10.3390/antiox13080985, PMID: 39199231 PMC11351922

[ref58] Mukherjee S. Chopra A. Karmakar S. Bhat S. G. (2025). Periodontitis increases the risk of gastrointestinal dysfunction: an update on the plausible pathogenic molecular mechanisms. Crit. Rev. Microbiol. 51, 187–217. doi: 10.1080/1040841X.2024.2339260, PMID: 38602474

[ref59] Nabavi-Rad A. Sadeghi A. Asadzadeh Aghdaei H. Yadegar A. Smith S. M. Zali M. R. (2022). The double-edged sword of probiotic supplementation on gut microbiota structure in *Helicobacter pylori* management. Gut Microbes 14:2108655. doi: 10.1080/19490976.2022.2108655, PMID: 35951774 PMC9373750

[ref60] Nagpal R. Mainali R. Ahmadi S. Wang S. Singh R. Kavanagh K.. (2018). Gut microbiome and aging: physiological and mechanistic insights. Nutr Healthy Aging 4, 267–285. doi: 10.3233/NHA-170030, PMID: 29951588 PMC6004897

[ref61] Nakagawa T. Lanaspa M. A. Millan I. S. Fini M. Rivard C. J. Sanchez-Lozada L. G.. (2020). Fructose contributes to the Warburg effect for cancer growth. Cancer Metab 8:16. doi: 10.1186/s40170-020-00222-9, PMID: 32670573 PMC7350662

[ref62] Neurath M. F. Artis D. Becker C. (2025). The intestinal barrier: a pivotal role in health, inflammation, and cancer. Lancet Gastroenterol. Hepatol. 10, 573–592. doi: 10.1016/S2468-1253(24)00390-X, PMID: 40086468

[ref63] Park R. Umar S. Kasi A. (2020). Immunotherapy in colorectal Cancer: potential of fecal transplant and microbiota-augmented clinical trials. Curr. Colorectal Cancer Rep. 16, 81–88. doi: 10.1007/s11888-020-00456-1, PMID: 32607098 PMC7325521

[ref64] Patel T. N. Roy S. Ravi R. (2017). Gastric cancer and related epigenetic alterations. Ecancermedicalscience 11:714. doi: 10.3332/ecancer.2017.714, PMID: 28144288 PMC5243136

[ref65] Pearson C. Uhlig H. H. Powrie F. (2012). Lymphoid microenvironments and innate lymphoid cells in the gut. Trends Immunol. 33, 289–296. doi: 10.1016/j.it.2012.04.004, PMID: 22578693

[ref66] Pepke M. L. Hansen S. B. Limborg M. T. (2024a). Telomere dynamics as mediators of gut microbiota–host interactions. Trends Cell Biol. 34, 805–808. doi: 10.1016/j.tcb.2024.08.003, PMID: 39256139

[ref67] Pepke M. L. Hansen S. B. Limborg M. T. (2024b). Unraveling host regulation of gut microbiota through the epigenome–microbiome axis. Trends Microbiol. 32, 1229–1240. doi: 10.1016/j.tim.2024.05.006, PMID: 38839511

[ref68] Pham V. T. Dold S. Rehman A. Bird J. K. Steinert R. E. (2021). Vitamins, the gut microbiome and gastrointestinal health in humans. Nutr. Res. 95, 35–53. doi: 10.1016/j.nutres.2021.09.001, PMID: 34798467

[ref69] Piscione M. Mazzone M. Di Marcantonio M. C. Muraro R. Mincione G. (2021). Eradication of Helicobacter pylori and gastric Cancer: a controversial relationship. Front. Microbiol. 12:630852. doi: 10.3389/fmicb.2021.630852, PMID: 33613500 PMC7889593

[ref70] Plamada D. Vodnar D. C. (2021). Polyphenols-gut microbiota interrelationship: a transition to a new generation of prebiotics. Nutrients 14:137. doi: 10.3390/nu14010137, PMID: 35011012 PMC8747136

[ref71] Porras A. M. Shi Q. Zhou H. Callahan R. Montenegro-Bethancourt G. Solomons N.. (2021). Geographic differences in gut microbiota composition impact susceptibility to enteric infection. Cell Rep. 36:109457. doi: 10.1016/j.celrep.2021.109457, PMID: 34320343 PMC8333197

[ref72] Rabaey K. Vandekerckhove T. de Walle A. V. Sedlak D. L. (2020). The third route: using extreme decentralization to create resilient urban water systems. Water Res. 185:116276. doi: 10.1016/j.watres.2020.116276, PMID: 32798895

[ref73] Rahman M. M. Islam M. R. Shohag S. Ahasan M. T. Sarkar N. Khan H.. (2022). Microbiome in cancer: role in carcinogenesis and impact in therapeutic strategies. Biomed. Pharmacother. 149:112898. doi: 10.1016/j.biopha.2022.112898, PMID: 35381448

[ref74] Rinninella E. Cintoni M. Raoul P. Lopetuso L. R. Scaldaferri F. Pulcini G.. (2019). Food components and dietary habits: keys for a healthy gut microbiota composition. Nutrients 11:2393. doi: 10.3390/nu11102393, PMID: 31591348 PMC6835969

[ref75] Salek Farrokhi A. Mohammadlou M. Abdollahi M. Eslami M. Yousefi B. (2020). Histone deacetylase modifications by probiotics in colorectal Cancer. J. Gastrointest. Cancer 51, 754–764. doi: 10.1007/s12029-019-00338-2, PMID: 31808058

[ref76] Sanches P. H. G. de Melo N. C. Porcari A. M. de Carvalho L. M. (2024). Integrating molecular perspectives: strategies for comprehensive multi-omics integrative data analysis and machine learning applications in transcriptomics, proteomics, and metabolomics. Biology (Basel) 13:848. doi: 10.3390/biology13110848, PMID: 39596803 PMC11592251

[ref77] Sandhu A. Chopra T. (2021). Fecal microbiota transplantation for recurrent Clostridioides difficile, safety, and pitfalls. Ther. Adv. Gastroenterol. 14:17562848211053105. doi: 10.1177/17562848211053105, PMID: 34992678 PMC8725027

[ref78] Sandoval-Motta S. Aldana M. Martinez-Romero E. Frank A. (2017). The human microbiome and the missing heritability problem. Front. Genet. 8:80. doi: 10.3389/fgene.2017.00080, PMID: 28659968 PMC5468393

[ref79] Servetas S. L. Daschner P. J. Guyard C. Thomas V. Affagard H. Sergaki C.. (2022). Evolution of FMT - from early clinical to standardized treatments. Biologicals 76, 31–35. doi: 10.1016/j.biologicals.2022.01.004, PMID: 35086768

[ref80] Sexton R. E. Al Hallak M. N. Diab M. Azmi A. S. (2020). Gastric cancer: a comprehensive review of current and future treatment strategies. Cancer Metastasis Rev. 39, 1179–1203. doi: 10.1007/s10555-020-09925-3, PMID: 32894370 PMC7680370

[ref81] Sgambato D. Miranda A. Romano L. Romano M. (2017). Gut microbiota and gastric disease. Minerva Gastroenterol. Dietol. 63, 345–354. doi: 10.23736/S1121-421X.17.02380-7, PMID: 28206729

[ref82] Shields H. J. Traa A. Van Raamsdonk J. M. (2021). Beneficial and detrimental effects of reactive oxygen species on lifespan: a comprehensive review of comparative and experimental studies. Front. Cell Dev. Biol. 9:628157. doi: 10.3389/fcell.2021.628157, PMID: 33644065 PMC7905231

[ref83] Shukla V. Singh S. Verma S. Verma S. Rizvi A. A. Abbas M. (2024). Targeting the microbiome to improve human health with the approach of personalized medicine: latest aspects and current updates. Clin Nutr ESPEN 63, 813–820. doi: 10.1016/j.clnesp.2024.08.005, PMID: 39178987

[ref84] Sipe L. M. Chaib M. Pingili A. K. Pierre J. F. Makowski L. (2020). Microbiome, bile acids, and obesity: how microbially modified metabolites shape anti-tumor immunity. Immunol. Rev. 295, 220–239. doi: 10.1111/imr.12856, PMID: 32320071 PMC7841960

[ref85] Szefel J. Danielak A. Kruszewski W. J. (2019). Metabolic pathways of L-arginine and therapeutic consequences in tumors. Adv. Med. Sci. 64, 104–110. doi: 10.1016/j.advms.2018.08.018, PMID: 30605863

[ref86] Takiishi T. Fenero C. I. M. Camara N. O. S. (2017). Intestinal barrier and gut microbiota: shaping our immune responses throughout life. Tissue Barriers 5:e1373208. doi: 10.1080/21688370.2017.1373208, PMID: 28956703 PMC5788425

[ref87] Tian H. Wang X. Fang Z. Li L. Wu C. Bi D.. (2024). Fecal microbiota transplantation in clinical practice: present controversies and future prospects. hLife 2, 269–283. doi: 10.1016/j.hlife.2024.01.006

[ref88] Tomela K. Pietrzak B. Schmidt M. Mackiewicz A. (2020). The tumor and host immune signature, and the gut microbiota as predictive biomarkers for immune checkpoint inhibitor response in melanoma patients. Life (Basel) 10:219. doi: 10.3390/life10100219, PMID: 32992737 PMC7600343

[ref89] Udayasuryan B. Zhou Z. Ahmad R. N. Sobol P. Deng C. Nguyen T. T. D.. (2024). *Fusobacterium nucleatum* infection modulates the transcriptome and epigenome of HCT116 colorectal cancer cells in an oxygen-dependent manner. Commun Biol 7:551. doi: 10.1038/s42003-024-06201-w, PMID: 38720110 PMC11079022

[ref90] Uebanso T. Shimohata T. Mawatari K. Takahashi A. (2020). Functional roles of B-vitamins in the gut and gut microbiome. Mol. Nutr. Food Res. 64:e2000426. doi: 10.1002/mnfr.202000426, PMID: 32761878

[ref91] Wang Y. Dong Q. Hu S. Zou H. Wu T. Shi J.. (2022). Decoding microbial genomes to understand their functional roles in human complex diseases. iMeta 1:e14. doi: 10.1002/imt2.14, PMID: 38868571 PMC10989872

[ref92] Wang P. Huang Q. Zhu Y. Chen L. Ye K. (2024). *Fusobacterium Nucleatum* promotes microsatellite instability in colorectal carcinoma through up-regulation of miRNA-155-5p-targeted inhibition of MSH6 via the TLR4/NF-κB signaling pathway. Adv Biol (Weinh) 8:e2400293. doi: 10.1002/adbi.202400293, PMID: 39334517

[ref93] Wang Y. Wang Y. Han W. Han M. Liu X. Dai J.. (2024). Intratumoral and fecal microbiota reveals microbial markers associated with gastric carcinogenesis. Front. Cell. Infect. Microbiol. 14:1397466. doi: 10.3389/fcimb.2024.1397466, PMID: 39355268 PMC11442432

[ref94] Wei Y. Gao L. Yang X. Xiang X. Yi C. (2022). Inflammation-related genes serve as prognostic biomarkers and involve in immunosuppressive microenvironment to promote gastric Cancer progression. Front Med (Lausanne) 9:801647. doi: 10.3389/fmed.2022.801647, PMID: 35372408 PMC8965837

[ref95] Wong C. C. Yu J. (2025). Pks+ *E. coli* adhesins—the fine line between good and evil. Cell Host Microbe 33, 1–3. doi: 10.1016/j.chom.2024.12.007, PMID: 39788090

[ref96] Wu S. Chen Y. Chen Z. Wei F. Zhou Q. Li P.. (2023). Reactive oxygen species and gastric carcinogenesis: the complex interaction between Helicobacter pylori and host. Helicobacter 28:e13024. doi: 10.1111/hel.13024, PMID: 37798959

[ref97] Wu J. Zhang R. Yin Z. Chen X. Mao R. Zheng X.. (2024). Gut microbiota-driven metabolic alterations reveal the distinct pathogenicity of chemotherapy-induced cachexia in gastric cancer. Pharmacol. Res. 209:107476. doi: 10.1016/j.phrs.2024.107476, PMID: 39490563

[ref98] Xie J.-Y. Ju J. Zhou P. Chen H. Wang S.-C. Wang K.. (2024). The mechanisms, regulations, and functions of histone lysine crotonylation. Cell Death Discov. 10:66. doi: 10.1038/s41420-024-01830-w, PMID: 38331935 PMC10853258

[ref99] Yang Y. Ke Y. Liu X. Zhang Z. Zhang R. Tian F.. (2024). Navigating the B vitamins: dietary diversity, microbial synthesis, and human health. Cell Host Microbe 32, 12–18. doi: 10.1016/j.chom.2023.12.004, PMID: 38211561

[ref100] Yang H. Wei B. Hu B. (2021). Chronic inflammation and long-lasting changes in the gastric mucosa after *Helicobacter pylori* infection involved in gastric cancer. Inflamm. Res. 70, 1015–1026. doi: 10.1007/s00011-021-01501-x, PMID: 34549319

[ref101] Yang J. Xu J. Wang W. Zhang B. Yu X. Shi S. (2023). Epigenetic regulation in the tumor microenvironment: molecular mechanisms and therapeutic targets. Signal Transduct. Target. Ther. 8:210. doi: 10.1038/s41392-023-01480-x, PMID: 37217462 PMC10203321

[ref102] Yang L. Ying X. Liu S. Lyu G. Xu Z. Zhang X.. (2020). Gastric cancer: epidemiology, risk factors and prevention strategies. Chin. J. Cancer Res. 32, 695–704. doi: 10.21147/j.issn.1000-9604.2020.06.03, PMID: 33446993 PMC7797232

[ref103] Yao W. Hu X. Wang X. (2024). Crossing epigenetic frontiers: the intersection of novel histone modifications and diseases. Signal Transduct. Target. Ther. 9:232. doi: 10.1038/s41392-024-01918-w, PMID: 39278916 PMC11403012

[ref104] Yao B. Wei W. Zhang H. (2025). Efficacy of probiotics or synbiotics supplementation on chemotherapy-induced complications and gut microbiota dysbiosis in gastrointestinal cancer: a systematic review and meta-analysis. Eur. J. Clin. Nutr. 79, 616–626. doi: 10.1038/s41430-024-01542-5, PMID: 39562823

[ref105] Yue B. Gao R. Wang Z. Dou W. (2021). Microbiota-host-irinotecan Axis: a new insight toward irinotecan chemotherapy. Front. Cell. Infect. Microbiol. 11:710945. doi: 10.3389/fcimb.2021.710945, PMID: 34722328 PMC8553258

[ref106] Zhang X. Pan Z. (2020). Influence of microbiota on immunity and immunotherapy for gastric and esophageal cancers. Gastroenterol Rep (Oxf) 8, 206–214. doi: 10.1093/gastro/goaa014, PMID: 32665852 PMC7333930

[ref107] Zhang Q. Schwarz D. Cheng Y. Sohrabi Y. (2024). Unraveling host genetics and microbiome genome crosstalk: a novel therapeutic approach. Trends Mol. Med. 30, 1007–1009. doi: 10.1016/j.molmed.2024.06.007, PMID: 38937208

[ref108] Zhang W. Xu L. Zhang X. Xu J. Jin J. O. (2023). *Escherichia coli* adhesion portion FimH polarizes M2 macrophages to M1 macrophages in tumor microenvironment via toll-like receptor 4. Front. Immunol. 14:1213467. doi: 10.3389/fimmu.2023.1213467, PMID: 37720226 PMC10502728

[ref109] Zhang X. Zhang Y. Wang C. Wang X. (2023). TET (ten-eleven translocation) family proteins: structure, biological functions and applications. Signal Transduct. Target. Ther. 8:297. doi: 10.1038/s41392-023-01537-x, PMID: 37563110 PMC10415333

[ref110] Zhao Y. Jiang Q. (2021). Roles of the polyphenol-gut microbiota interaction in alleviating colitis and preventing colitis-associated colorectal Cancer. Adv. Nutr. 12, 546–565. doi: 10.1093/advances/nmaa104, PMID: 32905583 PMC8009754

